# High content imaging shows distinct macrophage and dendritic cell phenotypes for psoriasis and atopic dermatitis

**DOI:** 10.1038/s41598-025-99727-w

**Published:** 2025-05-29

**Authors:** Nathalie J. Behr, Sandra Pierre, Tanja Ickelsheimer, Nicole Ziegler, Sonja Luckhardt, Aimo Kannt, Andreas Pinter, Gerd Geisslinger, Stephan M. G. Schäfer, Anke König, Klaus Scholich

**Affiliations:** 1 Institute of Clinical Pharmacology, Goethe University Frankfurt, Frankfurt, Germany; 2 Fraunhofer Institute for Translational Medicine and Pharmacology ITMP, Frankfurt, Germany; 3 Fraunhofer Cluster of Excellence for Immune-Mediated Diseases (CIMD), Frankfurt, Germany; 4 Institute of Dermatology, Venerology and Allergology, Hospital of the Goethe University, Frankfurt, Frankfurt, Germany

**Keywords:** High-content immunohistochemistry, Plaque psoriasis, Atopic dermatitis, Dendritic cells, Macrophages, T cells, Adaptive immunity, Imaging the immune system, Immunological disorders, Skin diseases

## Abstract

Psoriasis (Pso) and atopic dermatitis (AD) are chronic inflammatory skin diseases with distinct but also shared immunological features. Assessment of the feasibility of high content immunohistochemistry to identify distinct immune responses in skin biopsies from patients with chronic inflammatory skin diseases. While principal component analysis (PCA) based on the inflammatory marker profile discriminated healthy subjects from patients, it did not differ between Pso and AD. Single-cell phenotyping of high content immunohistochemistry images showed modest disease-specific differences for T cell populations in the number of Th1 T cells and γδT cells. Strong differences in macrophage and dendritic cell (DC) populations were observed whereby in AD disease-specific DCs with antiviral properties and anti-inflammatory macrophages were seen. Additional differences between Pso and AD were seen with the more frequent epidermal localization of CD8^+^ T cells in Pso and DCs in AD. Consequently, high content immunohistochemistry clearly discriminated AD from Pso based on macrophage and DC phenotype. Due to their phenotypic flexibility macrophages and DCs strongly reflect the immunological environment and serve to highlight disease-specific and -defining pathomechanisms and may serve to identify new drug targets.

**Supplementary Information:**

The online version contains supplementary material available at 10.1038/s41598-025-99727-w.

## Introduction

Atopic dermatitis (AD) and Plaque psoriasis (Pso) are multifactorial chronic inflammatory skin diseases with distinct immunological profiles. They are common diseases affecting between 2 and 3% of all adults for AD^1^and 1–2% for Pso^2^, although the prevalence varies with ethnicity and geographical region. In Pso Th1, Th17, and Th22 T cell subsets play a pathogenic role with involvement of various cytokines, including interferon (IFN)-γ, tumor necrosis factor (TNF)-α, interleukin (IL)-17 A, and IL-23^3^. Here, T cells of Type 17 (Th17, Tc17) are currently thought to be the main drivers of Pso^3^. AD is associated with a type 2 immune response including high levels of IL-4 and IL-13, and activation of Th2 and Th22 T cells leading to increased levels of IL-22 and IFNγ^4^. Fittingly, AD is often associated with an increased IgE production and allergies or asthma. Notably, some subtypes of AD have a prominent IL-17 component and histopathological similarities to Pso^1^. Thus, while Pso and AD share chronic inflammatory characteristics, their underlying immunologic processes involve different T cell responses, cytokine profiles, and immune cell activations. Understanding these differences is essential for the development of targeted and effective therapeutic approaches for each disease.

High content imaging based on sequential multiplex immunohistochemistry allows the visualization of an unlimited number of antibodies on the same tissue section^5,6^. This technology provides a new approach to identify and quantify immune and non-immune cells as well as their subpopulations in diseased tissue. At the same time the immediate neighbors of each identified cell are recorded and the data is used to identify region-specific cellular networks. Changes in these cellular networks can be used to identify basic pathomechanisms^7^, interpret responses to pharmacological interventions^8^, stratify patient groups^9^and predict patient outcome^10,11^. The technology is particularly useful to analyze the immune cell content of tissues, such as the skin epidermis, which are difficult to assess by standard methods, i.e. FACS analysis. Especially problems due to blood cells localized within vessels, but not within the tissue can be easily avoided by excluding blood vessels from the analysis^12,13^. However, a limitation of this technology is the time needed for runs with high antibody numbers, which can easily reach 2–3 days. As a consequence, it is necessary to perform initial discovery studies with high antibody numbers in a small patient population to determine the antibodies relevant for further studies in bioinformatics analyses. These initial discovery studies can then be followed by more diagnostic studies with fewer antibody numbers in a higher patient population. Here, we performed a discovery study to investigate the potential of high content immunohistochemical imaging to identify specific immune responses in patients with Pso or AD.

## Results

### Multiplex analysis of inflammatory markers does not distinguish Pso and AD

**Fig. 1:**
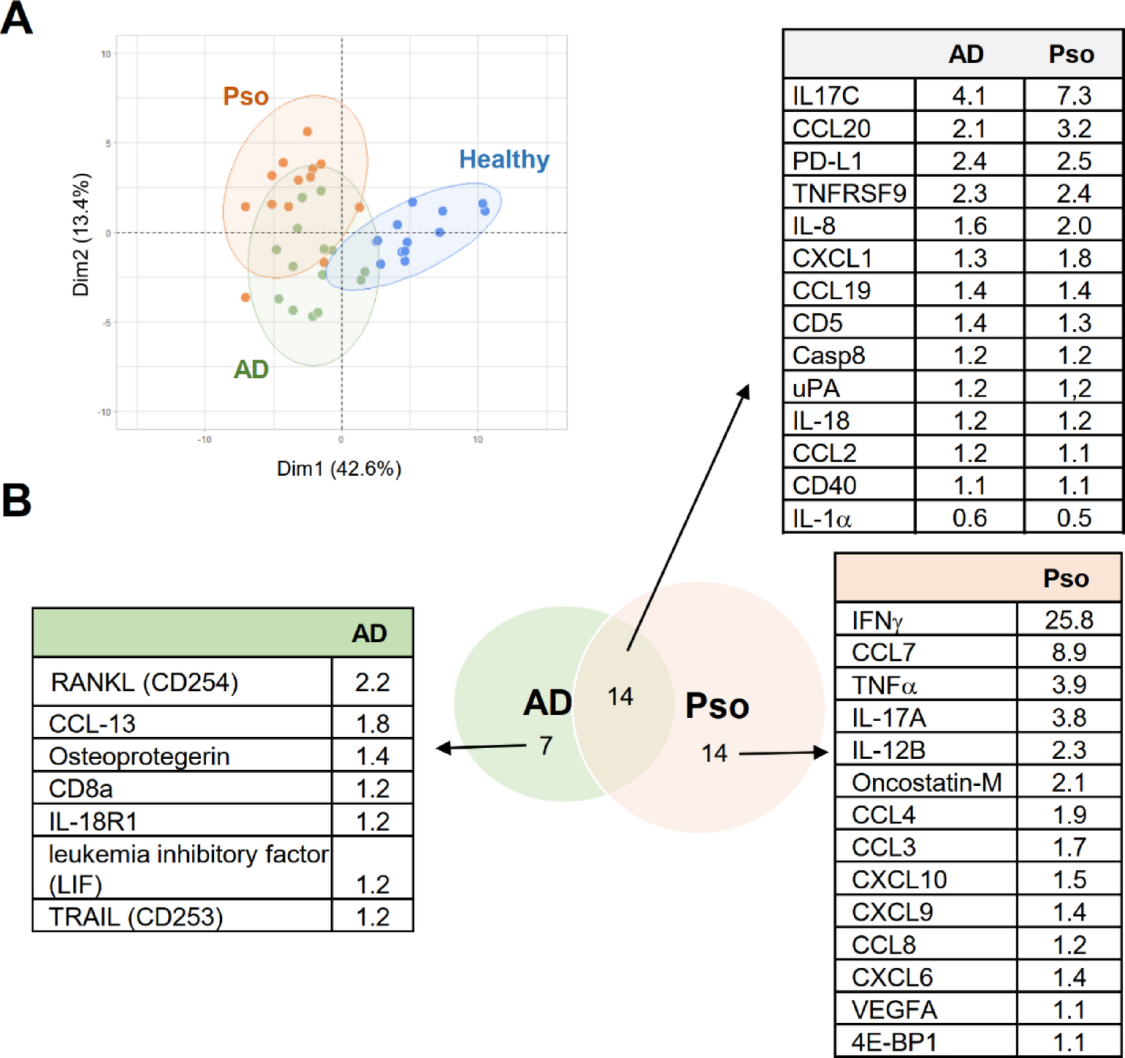
Differential analysis of inflammation marker levels in skin biopsies of Pso and AD patients. **(a)** Principal component analysis of inflammation marker expression using the Olink^®^ platform of skin biopsies from healthy participants (*n* = 13) and patients with Pso (Pso; *n* = 13) or AD (*n* = 14). **(b)** Fold change of expression of significantly up- or downregulated inflammation markers (Olink^®^) in Pso and AD patients as compared to healthy participants.

To compare the immune responses in Pso and AD we first determined the expression of 92 inflammation markers using the Olink^®^proteomics platform. Here, skin biopsies of 14 Pso and 14 AD patients were compared. As controls, 13 biopsies from healthy donors were used. All participants were previously analyzed for their plasma metabolom and lipidome^14^. The patients comprised the whole spectrum of the disease scores ranging in each disease group from mild to severe (Pso: PASI score 5.4–32.5; AD: EASI score 4.7–29.1; supporting information Table S1)^14^. In total 70 of the 92 investigated proteins were detectable in at least one of the three groups, whereby 35 markers showed a significant up- or down-regulation in at least one of the groups as compared to healthy subjects (supporting information Table S2). Principal component analysis separated the samples from patients and healthy donors based on these 35 inflammation markers but did not distinguish between Pso and AD (Fig. 1A). Pso and AD shared a similar regulation of 14 inflammatory markers, while 14 markers were Pso-specific and 7 AD-specific (Fig. 1B and supporting information Table S2). As expected, prototypical proinflammatory mediators IL-17 A, IFN-γ, TNF, VEGF-A, CCL7 and IL-12B, which are known to promote disease progression in Pso^15,16^, were significantly upregulated in Pso but not in AD (Fig. 1B). In addition, several chemokines (CCL7, CCL4, CCL3, CCL8, CXCL10, CXCL9 and CXCL6) were specifically regulated in Pso, which are known for targeting leukocytes or to induce Th1 and Th17 polarization. In AD biopsies only 5 markers were specifically upregulated, which included several mediators involved in cell differentiation and survival (RANKL, osteoprotegerin, leukemia inhibitory factor (LIF) and TRAIL) (Fig. 1B and supporting information Table S2). Importantly, among the AD-specific markers were the CCR1 ligands CCL13 and LIF), which can inhibit proinflammatory responses in macrophage and DC^17^. Taken together, the data emphasize the different immune responses underlying AD and Pso, but also show difficulties in distinguishing both diseases in principal component analysis due to a large overlap in the regulation of immune mediators.

### Antiinflammatory macrophage phenotypes are more abundant in AD than in Pso

**Fig. 2:**
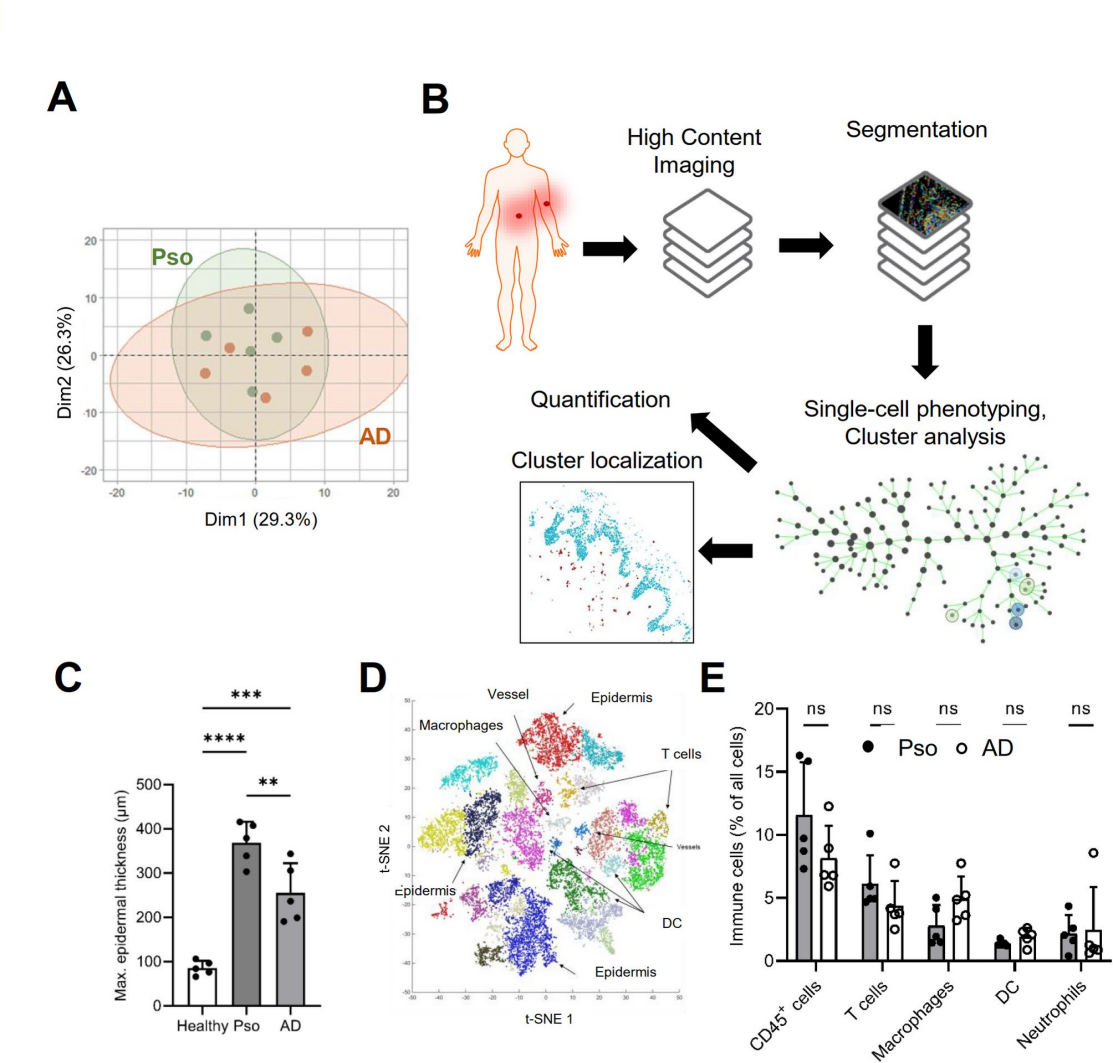
Comparison of patients included in the MELC discovery study. **(a)** Principal component analysis of inflammation marker expression using the Olink^®^ platform of skin biopsies from patients with Pso (Pso; *n* = 5) or AD (*n* = 5). **(b)** Workflow of the bioinformatic analysis for the MELC images. **(c)** Maximal epidermal thickness of the healthy participants and Pso or AD patients. Data are shown as mean ± S.E.M. (*n* = 5). One Way ANOVA, Bonferroni posthoc test, **p < 0.01, ***p < 0.001. **(d**,** e)** Representative t-SNE plot (panel d) and quantification (panel e) of the major immune cell populations in the skin biopsies detected in MELC images. Data are shown as mean ± S.E.M. (*n* = 5). Two tailed Student’s t-test (ns, not significant).

Since Pso and AD were not clearly separated in the principal component analysis based on the expression of inflammatory markers, we tested whether high content imaging analysis of the biopsies could differentiate between the two diseases. To avoid a bias in the selection of patient samples for MELC analysis biopsies from 5 Pso patients and 5 AD patients were selected for imaging, which show similar inflammation marker expression patterns, as shown by the homogeneous distribution in the principal component analysis (Fig. 2A). The biopsies were subjected to high-content MELK imaging using 41 antibodies, which allow to identify important immune and non-immune cells as well as their subtypes (supplementary information Table S3). The visual fields were selected to cover an area from stratum corneum to the dermis (Fig. 2B). A segmentation mask was generated based on nuclear staining and was used to extract single-cell expression data from the images of all markers. Single-cell phenotyping (PhenoGraph analysis) was followed by the identification of cell clusters representing the different immune cell types, which then can be quantified and their localization visualized using histoCAT v1.76^18^ (Fig. 2B). For better clarity we will mention in the following only the relevant markers, which were used to define specific cell types. Epidermal thickness, as measured by the maximum distance between *stratum corneum* and *stratum basale*, was significantly increased in both diseases, although the epidermal thickening was more pronounced in Pso (Fig. 2C). The number of all immune cells, as determined by the percentage of CD45^+^ cells of all cells in the tissue, did not differ between the two groups in the areas observed (Fig. 2D, E). Similarly, there were no significant differences between Pso and AD in the cell count of clusters describing either all T cells (CD3^+^), monocytes/macrophages (CD14^+^), dendritic cells (DCs; CD11c^+^/CD14^-^) or neutrophils (CD66b^+^). B cells, or NK-cells were not regularly detected or were present in such low numbers that they could not be identified as independent cell clusters.

**Fig. 3:**
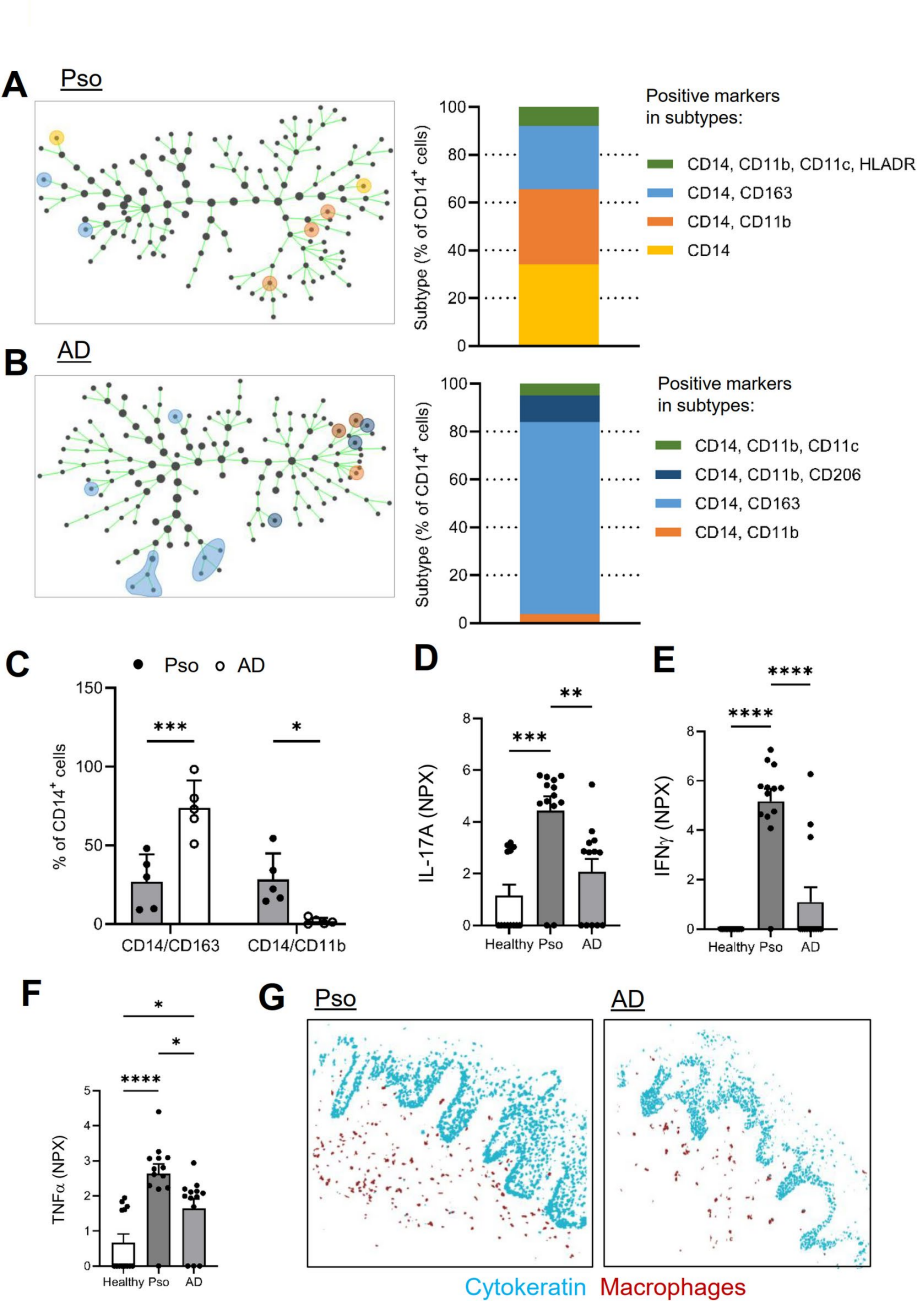
Macrophage subpopulations show disease-specific variations. **(a**,** b)** SPADE cluster analysis of all cells (left). Macrophage subpopulations are depicted by colored circles in Pso (panel a) and AD (panel b) patients. The same color is used for a subtype in all panels. The percentage of the macrophage populations (right) based on the MELC analysis. **(c)** Quantification of the number of CD163^+^ and CD11b^+^ macrophages in Pso and AD patients. Data are shown as mean ± S.E.M. (*n* = 5). Two tailed Student’s t-test, *p < 0.05, ***p < 0.001. **(d-f)** Tissue expression of IL-17 A (panel d), IFNγ (panel e) and TNFα (panel f) determined by OLINK^®^ in skin biopsies of Pso and AD patients as well as healthy volunteers. Data are shown as mean ± S.E.M. (*n* = 5). One-way ANOVA, *p < 0.05, **p < 0.01, ***p < 0.001. **(g)** Representative images showing clusters containing cytokeratin (light blue) and clusters representing CD14^+^ macrophages (brown).

Detailed analysis of the CD14^+^ cell clusters showed strong differences in the macrophage subpopulations between Pso and AD. In Pso, three macrophage clusters were observed. 65% of all CD14^+^ cells were not clearly differentiated macrophages (CD14^+^/CD11b^-/+^/CD163^-^/CD11c^-^/CD1c^-^/HLADR^-^). 25% of all CD14^+^ cells were anti-inflammatory macrophages (CD14^+^/CD11b^-^/CD163^+^/CD11c^-^/CD1c^-^/HLADR^-^) and 10% of all CD14^+^ cells were monocyte-derived macrophages (CD14^+^/CD11b^+^/CD163^-^/CD11c^+^/CD1c^-^/HLADR^-/+^) (Fig. 3A). Importantly, in AD approximately 90% of all CD14^+^ cells were anti-inflammatory macrophages (CD14^+^/CD11b^-^/CD163^+^ and CD14^+^/CD11b^+^/CD206^+^), while other macrophage subpopulations appeared only sparsely (Fig. 3B, C). The finding that macrophages with anti-inflammatory properties dominate the inflamed tissue in AD is consistent with lower levels of pro-inflammatory mediators (e.g. IFN-γ, IL-17 A, and TNF-α) in AD as compared to Pso (Fig. 3D-F). In both Pso and AD, regardless of their phenotype, all CD14^+^ cells were located almost exclusively in the dermis and not in the epidermis. (Fig. 3G).

### CD123^+^ and CCR4^+^ DCs are present in AD but not Pso

**Fig. 4:**
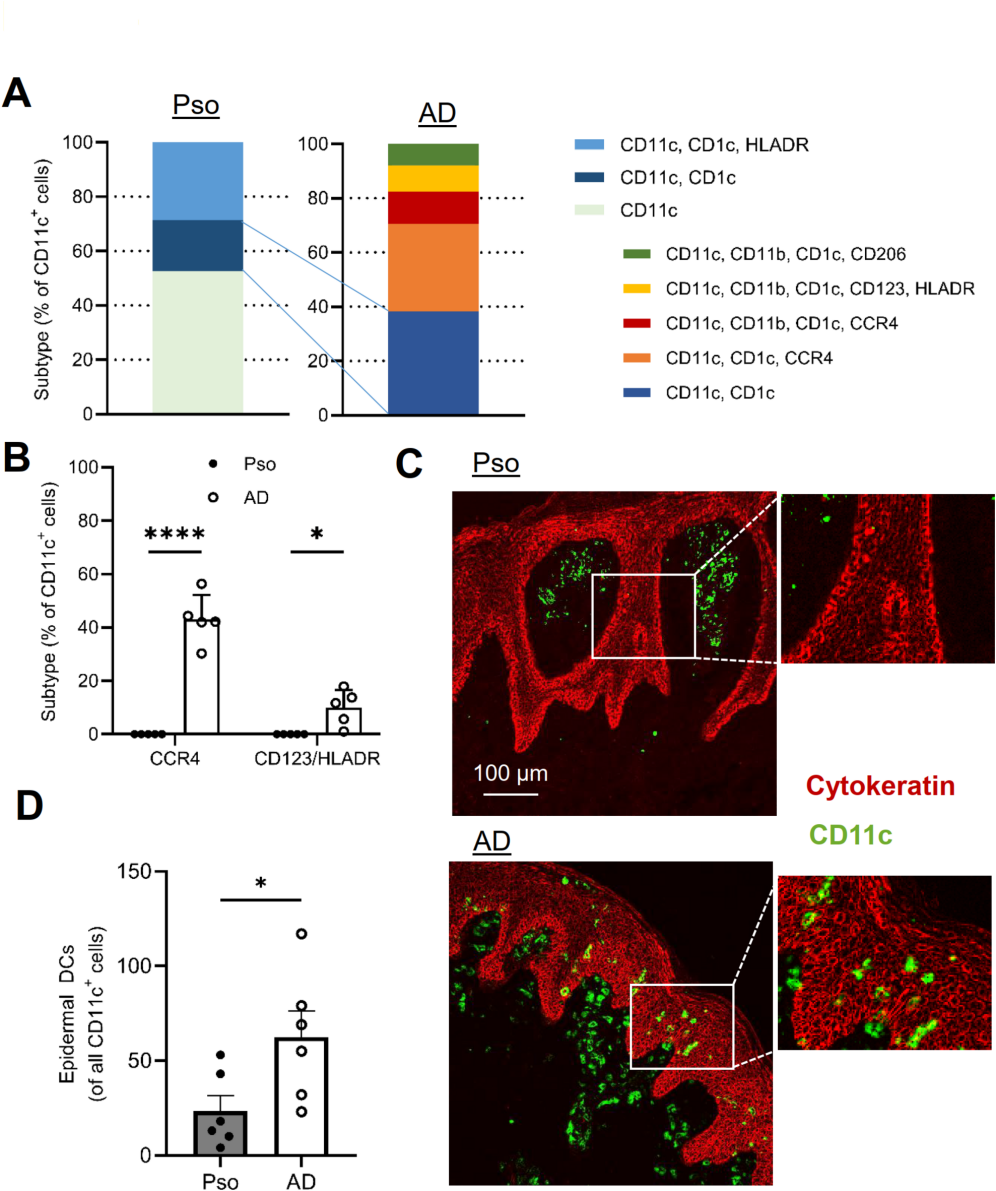
DCs show disease-specific subpopulations and localization. **(a)** Percentage of the DC populations as % of all DCs in skin biopsies of Pso and AD patients. The same color is used for a subtype in all panels. **(b)** Quantification of the number of CCR4 or CD123 expressing DCs in Pso and AD patients. Data are shown as mean ± S.E.M. (*n* = 5). Two tailed Student’s t-test, *p < 0.05, ****p < 0.0001. **(c**,** d)** Quantification (panel c) and representative images (panel d) showing epidermal localization of DCs. Data are shown as mean ± S.E.M. (*n* = 5). Two tailed Student’s t-test, *p < 0.05.

Similar to macrophages, also dendritic cells (CD11c^+^/CD14^-^) exhibited in both diseases distinct DC subpopulations. Three major DC subpopulations were observed in Pso, with 52% of these cells being CD11c^+^/CD1c^-^/CD11b^-^/HLADR^-^ DCs, 19% were CD11c^+^/CD1c^+^/CD11b^-^/HLADR^-^ DC1-type cells and 29% were CD11c^+^/CD1c^+^/CD11b^-^/HLADR^+^ DC2-type cells (Fig. 4A). Out of these 3 populations only the DC1-type DCs were also seen in AD biopsies representing 35% of all DCs (Fig. 4A). In addition, AD biopsies included a previously not clearly defined CD11c^+^/CCR4^+^subpopulation with antiviral properties^19^ and CD11c^+^/CD123^+^plasmacytoid-type DCs (pDCs), which are capable to produce type 1 interferons in response to viral infections^20,21^. Here, the CCR4-positive population comprised 44% of all DCs (CD11c^+^/CD1c^+^/CD11b^-/+^/CCR4^+^/HLADR^-^) and the CD123-positive population (CD11c^+^/CD1c^+^/CD11b^+^/CD123^+^/HLADR^+^) around 10% of all DCs (Fig. 4A, B). A further 8% of all DCs were CD11c^+^/CD1c^+^/CD11b^+^/CD206^+^/HLADR^-^DCs, which were described earlier as tolerogenic DCs^22,23^. Thus, the three subpopulations expressing CCR4, CD123 or CD206 represented together around 64% of all DCs and are associated with antiviral and tolerogenic immune responses^19–23^.

Regarding the localization of DCs expressing CD11c and/or CD1c, we found that they are located mainly in the dermis in both diseases with rare infiltration of the epidermis in AD and Pso. In Pso epidermal DCs were present in relative low numbers (7% of all DCs) and appeared to be randomly distributed throughout the epidermis (Fig. 4C, D). In AD, we observed significantly higher numbers of DCs in the epidermis, which often appeared in clusters in some epidermal areas, and represented 13.5% of all DCs (Fig. 4C, D). In conclusion, Pso and AD show strong differences in their DC subpopulations, representing different immune responses, as well as in the DC infiltration of the epidermis.

### T-cell localization and differentiation differ between AD and Pso

**Fig. 5:**
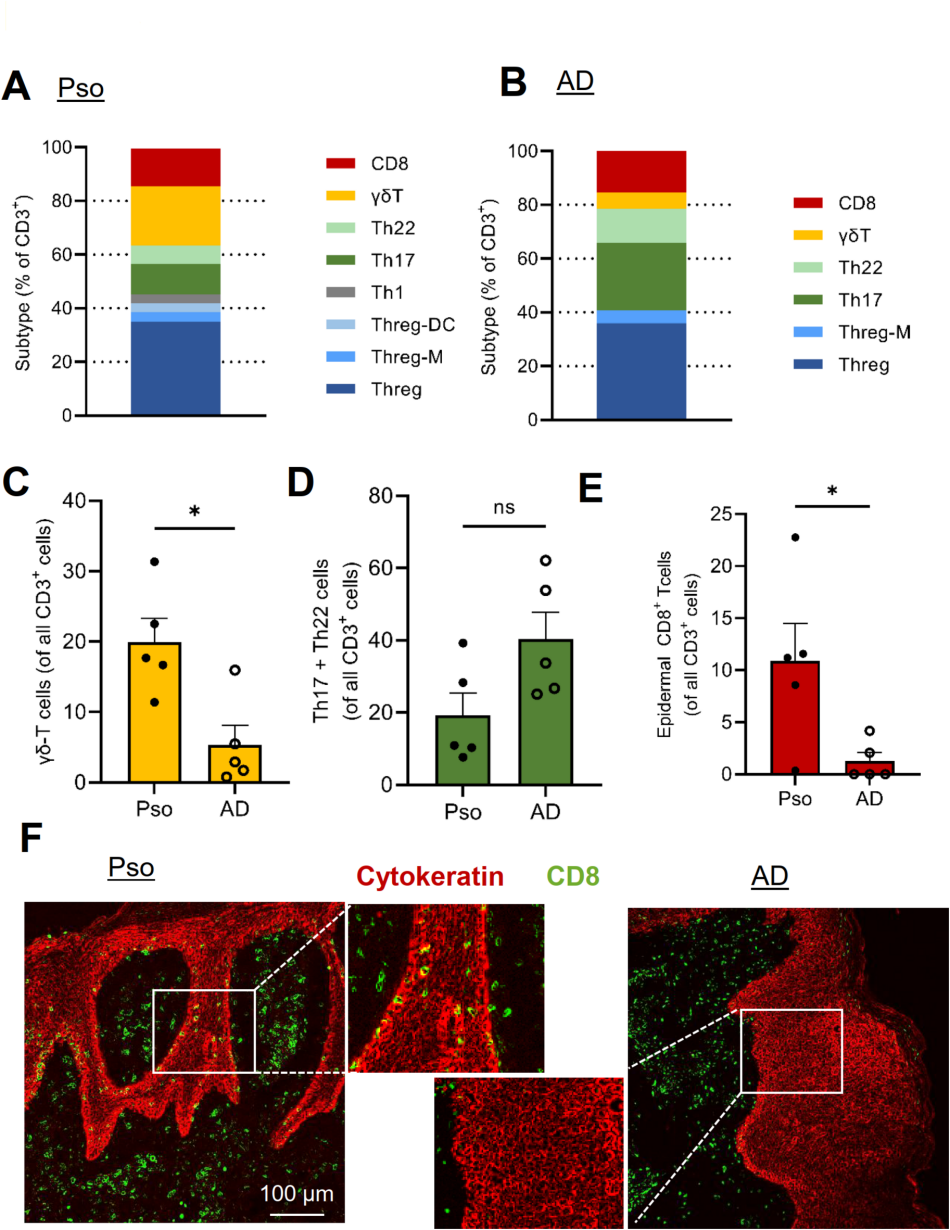
T cells show disease-specific subpopulations and localization. **(a**,** b)** Quantification of T cell populations in Pso (panel a) and AD (panel b) patients. The percentage of the T cell populations is shown as total number of T cells (right). Each subpopulation is depicted by the same color in all panels. **(c**,** d)** Quantification of the number of γδT cells (panel c) and Th17/Th22 T cells (panel d) in Pso and AD patients. Data are shown as mean ± S.E.M. (*n* = 5). Two tailed Student’s t-test, *p < 0.05, **p < 0.001. **(e**,** f)** Quantification (panel e) and representative images (panel f) showing epidermal localization of CD8a T cells. Data are shown as mean ± S.E.M. (*n* = 5). Two tailed Student’s t-test, *p < 0.05.

T-cell subpopulations observed in Pso and AD differed to a lesser extent between the diseases than seen for DC and macrophage populations. Cell clusters for Threg, Th17, Th2/22, γδ-T cells and CD8 T cells were present in both diseases, whereas Th1-type T cells were only detected in Pso (Fig. 5A, B; Supplemental Table S4). Other major differences in cell numbers were a higher percentage of γδ-T cells in Pso (Pso: 20%, AD: 4.5%) and in AD more Th17, and Th2/Th22 cells (Pso: 7%, AD 13%) (Fig. 5C, D). The number of CD8 T cells did not differ between both diseases. However, CD8 T cells are known to infiltrate the epidermis in Pso but not in AD^24,25^ and consistent with this notion, 10.8% of all CD8-positive cells were found in the epidermis in Pso, whereas in AD biopsies only 1% of all CD8-positive cells were located in the epidermis (Fig. 5E, F). Taken together, Pso and AD show clear but less dramatic differences in their T cell subpopulations as compared to DC and macrophage populations.

**Fig. 6:**
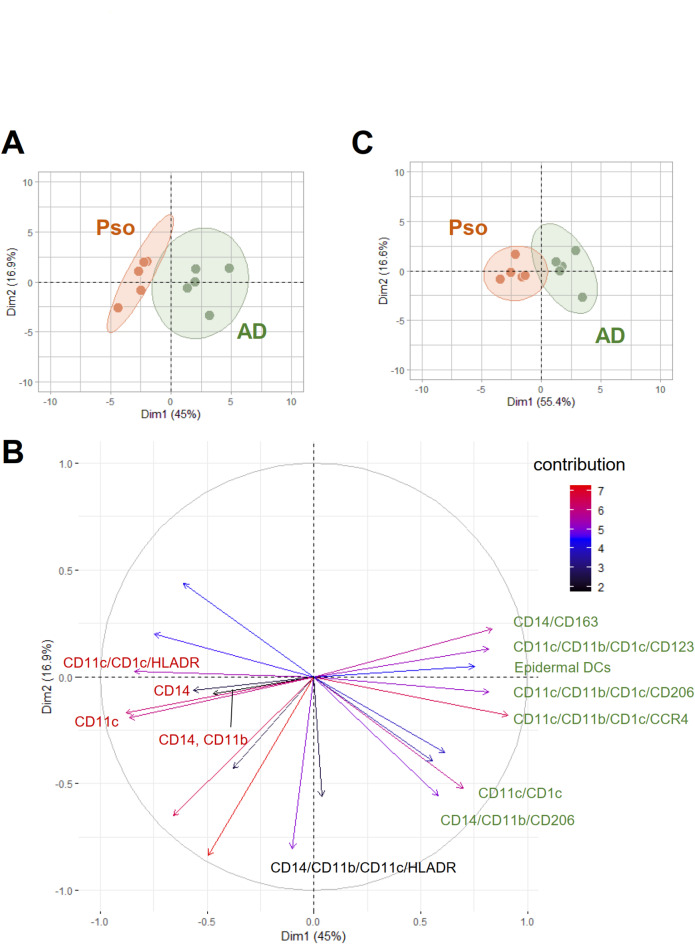
Principal component analysis based on the MELC analysis differentiates between Pso and AD patients. **(a)** Principal component analysis of macrophage, DC and T cell populations as well as epidermal DC and CD8a^+^ T cell localization of skin biopsies from patients with Pso (*n* = 5) or AD (*n* = 5). **(b)** Same as panel a, except that the contribution of each cluster to principal component analysis is shown. Only DC and macrophage populations are labeled. See supplemental data S4 for complete labels. **(c)** Same as panel a, except that only the data for DC and macrophage subtypes were used. The list of markers used for this principal component analysis are marked in bold supplemental data S2.

Principal component analysis based on immune cell clusters, which include the different DC, macrophage and T-cell subtypes, and epidermal localization data for DCs and CD8^+^ T cells, showed a clear distinction between Pso and AD patients (Fig. 6A). A detailed analysis of the contribution of the different cell types showed a strong contribution of macrophage and DC phenotypes (Fig. 6B and Supplemental data 5). Accordingly, reduction of the analyzed markers to 9 antibodies (Supplemental data S4), which describe the DC and macrophage subtypes, was sufficient to distinguish both diseases (Fig. 6C).

## Discussion

We used high content immunohistochemistry in a discovery study to identify the subpopulations of T cells, macrophages and DCs, which are present in the skin tissue of AD and Pso patients. We found especially strong differences between the diseases in regard to macrophage and DC phenotypes, which underline the differences between both diseases. Due to their phenotypical flexibility macrophages and DCs are especially well suited to reflect the immunological environment and can highlight disease-specific and -defining pathomechanisms. The clear differences between both diseases in our study are surprising because all included patients varied widely in disease severities. Importantly, since the patients showed similar total CD45^+^ cell numbers in the biopsies, the data suggest that similar local quantitative and qualitative disease activities can be found in patients despite differences in clinical scores.

Comparison of the expression profiles of inflammatory markers in the skin biopsies from our Pso and AD patients showed that 66% of all regulated markers were similarly up- or downregulated in both diseases. The overlapping immune marker regulation reflects common stress and immune responses. Several markers showed a differential regulation between AD and Pso patients, whereby the proteomics data do not contain the spatial information, such as in which cell types a differentially regulation occurs, that can be obtained by using MELC. Characterization and quantification of the immune cells and their subtypes showed strong differences particularly between macrophage and DC phenotypes as well as their epidermal localization. DC populations were in Pso largely DC1 and DC2 populations, whereas in AD CCR4, CD206 and CD123-expressing DC subpopulationss were seen, which are associated with antiviral and tolerogenic functions^19–23^. Fittingly, the macrophage population in AD consisted mainly out of CD163 or CD206 expressing macrophages, which are associated with anti-inflammatory functions. Macrophages and DCs are known to adapt their gene expression patterns flexible to the signals they receive from their microenvironments, leading to the formation of distinct subpopulations.

The differences of the macrophage and DC subtypes between the diseases were unexpected in its extent. Notably, the prototypical proinflammatory markers TNFα, IFNγ and Il-17 A were only significantly increased in Pso supporting the known Th1/Th17-type response in Pso patients^3^. Although Pso and AD are known to depend on Th17 cells, a significant increase of IL-17 A as compared to biopsies from healthy donors was only observed in Pso, which is in accordance with the therapeutic success of anti-IL-17 A treatments in this disease^26^. Several cell types, including Th17, Th22, γδT cells and CD8 T cells, were detected in Pso and AD, which have been reported previously to be potentially able to produce IL-17 A. The obvious discrepancy between the observed presence of these immune cells capable to produce IL-17 A and the levels of IL-17 A in AD underlines that the presence of these cells alone cannot automatically relate to the production or release of IL-17 A. IL-4 and IL-13 are likely candidates to explain the macrophage and DC phenotypes in AD, since they are able to induce the observed alternative activated phenotypes^27,28^and are known to play a major role in the immunopathology of AD^4^. Fittingly, biologicals directed against IL-4 and IL-13 are approved drugs for the therapy of AD^29^.

Finally, a clear separation of Pso and AD biopsies can be achieved based on T cell, DC and macrophage subtypes together with their localization. However, due to the relative low numbers of immune cells invading the epidermis, the distinction between the diseases based on the phenotypes of macrophages and DCs instead of the localization seems more reliable and easier to define.

It should be noted that this pilot study is limited by the low number of patients included in the analysis. Therefore, additional disease-specific differences might be observed when additional patients are included in the analysis, which might also allow to determine score-dependent or sex-specific differences in the immune responses. However, the currently available technical systems suffer by the time-consuming measurements, restricting either the number of patients or the number of antibodies used. Pilot studies such as the study presented here help to identify interesting markers, which then can be further validated in larger patient groups. Thus, since reducing the analysis to DCs and macrophages markers is sufficient to distinguish both diseases, the reduction on the set of 9 antibodies will allow screening of higher biopsy numbers as well as faster subtype analysis, based on a standardized preestablished bioinformatic workflow^30^.

In conclusion, the characterization of Pso and AD biopsies by high-content imaging based on multiple immunohistochemistry shows that this technology is a valuable tool to detect disease-specific immune cell responses. The focus on macrophage and DC subtypes as biomarkers and an expansion of markers distinguishing even more subpopulations of both cell types, might present an approach to identify the patient-specific pathomechanisms underlying these two chronic skin diseases and might also help to choose the most promising therapy options.

## Methods

### Study design

The study was conducted in accordance with the Helsinki Declaration principles. Use of human materials was approved by the institutional ethics committee (Ethik-Kommission des Fachbereiches Medizin, Klinikum der Goethe Universität, business number 184/19, ethics vote 73/19). All participants were adults and provided written informed consent for the use of their samples. This study included 27 patients with either active psoriasis (*n* = 13) or AD (*n*= 14) for at least six months. Additionally, a control group of 13 healthy volunteers were recruited. Lipidomic and metabolomic analyses of blood samples from all study participants have been published previously^14^. Exclusion criteria for all subjects were recent or current use of local or systemic immune suppressants, drug abuse and pregnancy. Disease severity scores for the respective diseases were determined using the “Pso area and severity index” (PASI) and the “Exzema area and severity index” (EASI). Medical history and medical or surgical treatments prior to study enrollment were recorded^14^. The patients were recruited during special consultations for the respective disease at the Department of Dermatology, Venereology, and Allergology of the University Hospital Frankfurt. Biopsies of patients were taken in acutely inflamed body areas, while biopsies of healthy volunteers were taken at comparable locations. One biopsy was fixed in Tissue-Tek O.C.T. Compound (Sakura Finetek Umkirch, Germany) and stored at -80 for MELC analysis, while the second sample was frozen without fixing for Olink^®^ measurements.

### Epidermal thickness

The maximum distance between the stratum corneum and stratum basale was measured in 10 μm frozen tissue sections using Fiji 2.16.0 to determine the epidermal thickness. For each patient five different areas in the field of vision were chosen to depict a precise average value for the epidermal thickness.

### Inflammatory markers determined by Olink^®^

Biopsies were lysed in 100 µl RIPA buffer (50 mM Tris-HCl pH 7.4, 150 mM NaCl, 1 mM EDTA from Merck, Darmstadt, Germany) with 1× Protease Inhibitor Cocktail (Roche, Mannheim, Germany). Samples were sonicated 3x at 70% power for 10 s using a SONOPULS HD2070 MS73 (Bandelin, Berlin, Germany). After centrifugation (1300 x g, 5 min) the total protein concentration in the supernatant was determined using a NanoDrop UV/VIS spectrophotometer (Thermo Fisher Scientific). All samples were adjusted to a final protein concentration of 1 mg/ml. Expression of inflammatory markers was determined in 1 µl using the Olink^®^ Target 96 Inflammation panel as recommended by the manufacturer (Olink^®^, Uppsala, Sweden) using a Biomark HD (Standard Biotools, South San Francisco, CA, USA). Assay QC and data processing was performed using the NPX^™^ Signature Software (Olink^®^, Uppsala, Sweden). Data is presented as NPX values (Normalized Protein eXpression), Olink^®^’s arbitrary unit in Log2 scale.

### High-content immunohistochemistry

Multi-epitope-ligand cartography (MELC) is an automated immunohistological imaging method that can be used to visualize high numbers of antibodies on the same sample^6,7^. Briefly, 10 μm tissue sections on silanized coverslips were fixed with 4% paraformaldehyde in PBS for 10 min, permeabilized with 0.1% Triton X-100 in PBS for 10 min, and blocked with 3% bovine serum albumin (all from Merck, Darmstadt, Germany) in PBS for 1 h. Tissue samples were imaged using a DMi8 microscope (Leica Microsystems, Wetzlar, Germany) and a HC PL FLUOTAR L 20x/0,040 CORR PH1 objective with a cooled sCMOS camera (2048 × 2048 pixels). The sample was then incubated with up to 3 antibodies, each labelled with different bleachable fluorescence-tags, and washed with PBS. Phase-contrast and fluorescence images were collected, the fluorescence signals were bleached and post-bleaching images were recorded. The process was repeated until all antibodies were imaged. For data analysis, the post-bleaching images were subtracted from the following fluorescence image. The antibodies used on the MELC system are listed in the Supporting Information Table S1.

### Image analysis

All grayscale antibody channel images were processed using ImageJ v1.52 (NIH, Bethesda, MD, USA) to remove noise, background fluorescence and artifacts. Cell Profiler v4.2.1^31^was used for additional illumination correction and the generation of a cell mask for single-cell segmentation using the propidium iodide (nuclei) signals. The segmentation mask was imported into histoCAT v1.76^18^with the corresponding antibody channel images. All images, excluding those used for single-cell mask generation, were z-score normalized and used for Barnes-Hut (BH) t-SNE^32^and PhenoGraph analysis^33^as implemented in histoCAT. PhenoGraph defines cell clusters based on single-cell mask and marker co-localization (k = 10). Single-cell segmented datasets from histoCAT clusters were exported as CSV files, converted to FCS format and analyzed with Spanning-trees of density-normalized events (SPADE) v3.0 in MATLAB vs2023a to generate spanning trees of density-normalized events under standard conditions (k-means for 150 desired clusters)^34^. Clusters were classified as different cell types based on marker expression. The number of objects per cluster was normalized to the total number of objects in the cell mask to calculate the relative number of cells per cell type.

### Statistical analysis

Data processing and statistical analysis were performed in R v4.3.2 (2023 − 10 − 31) (RStudio, Boston, MA, USA) using RStudio programming interface 2023.12.1.402 64-bit (R Foundation for Statistical Computing, Vienna, Austria) including the R packages “readxl” v1.4.3, “factoextra” v1.0.7, “ggplot2” v3.5.1 and “dplyr” v1.1.4. Statistically significant differences were determined by one-way ANOVA followed by *post hoc* Bonferroni correction for multiple comparisons. For comparisons between two groups, we used an unpaired two-tailed Student’s *t-*test.

## Electronic supplementary material

Below is the link to the electronic supplementary material.

Supplementary Material 1

## Data Availability

The data underlying this article are available in the article and the supplementary information.

## Abbreviations

AD: Atopic Dermatitis
CD: cluster of differentiation
CCL: C-C chemokine motif ligand
IFN: interferon
IL: Interleukin
MELC: Multi-Epitope-Ligand Cartography
Pso: Psoriasis
Th: T helper cell
TNFα: Tumor necrosis factorα
