# Supplementary material for: High content imaging shows distinct macrophage and dendritic cell phenotypes for psoriasis and atopic dermatitis
Source: Sci Rep. 2025 May 29;15:18904. doi: 10.1038/s41598-025-99727-w (PMC12122870; doi:10.1038/s41598-025-99727-w)
Supplement: Supplementary file 1 — Supplementary Material 1 [file 41598_2025_99727_MOESM1_ESM.pdf]

High content imaging shows distinct macrophage and dendritic cell phenotypes for psoriasis and atopic dermatitis

Nathalie J. Behr, Sandra Pierre, Tanja Ickelsheimer, Nicole Ziegler, Sonja Luckhardt, Aimo Kannt, Andreas Pinter, Gerd Geisslinger, Stephan MG Schäfer, Anke König, Klaus Scholich

Supplementary Table S1:

Table S1: Patient characteristics

Patienten-ID	Age (years)	Disease	Sex	Height (cm)	Weight (kg)	Biopsy location	DLQI	EASI-Score	disease severity
3	26	Atopic Dermatitis	Female	161	73	arm	18	15	moderate
7	23	Atopic Dermatitis	Female	160	65	leg	4	4,7	mild
19	29	Atopic Dermatitis	Male	180	64	Abdomen	21	17,4	moderate
22	21	Atopic Dermatitis	Female	166	80	back	24	15,7	moderate
24	30	Atopic Dermatitis	Female	167	80	leg	23	29,1	severe
28	30	Atopic Dermatitis	Male	169	55	neck	19	10,2	moderate
29	30	Atopic Dermatitis	Female	162	60	arm	15	14	moderate
30	36	Atopic Dermatitis	Male	191	95	leg	16	22,8	severe
31	19	Atopic Dermatitis	Female	170	60	leg	20	15,3	moderate
32	25	Atopic Dermatitis	Male	185	75	back	22	13,4	moderate
34	43	Atopic Dermatitis	Male	180	102	back	8	9	moderate
35	20	Atopic Dermatitis	Female	173	68	back	15	11,5	moderate
36	20	Atopic Dermatitis	Male	192	73	back	9	18	moderate
40	28	Atopic Dermatitis	Female	167	62	arm	28	16	moderate
41	30	Atopic Dermatitis	Male	179	80	back	9	46,3	severe

Patienten-ID	Age (years)	Disease	Sex	Height (cm)	Weight (kg)	Biopsy location	DLQI	PASI-Score	disease severity
2	47	Psoriasis	Male	202	101	leg	1	10,7	moderate
6	26	Psoriasis	Male	182	100	leg	1	10,1	moderate
8	46	Psoriasis	Female	173	65	hip	10	10,5	moderate
9	26	Psoriasis	Male	182	95	back	0	20,4	moderate
11	56	Psoriasis	Male	174	108	Abdomen	0	11,1	moderate
14	56	Psoriasis	Male	171	122,4	back	22	14,4	moderate
15	29	Psoriasis	Male	180	120	Abdomen	28	32,5	moderate
16	33	Psoriasis	Male	183	85	back	13	13,8	moderate
17	49	Psoriasis	Male	179	102	Abdomen	0	5,4	mild
18	32	Psoriasis	Female	169	157	Abdomen	17	16,7	moderate
19	27	Psoriasis	Female	164	62	leg	11	7,7	mild
23	31	Psoriasis	Male	180	95	leg	11	9,8	mild
25	28	Psoriasis	Male	188	110	back	3	5,8	mild
26	37	Psoriasis	Male	186	105	Abdomen	6	7,2	mild
27	18	Psoriasis	Male	181	104	back	5	12,6	moderate

Patients marked in brown were included in the MELC analysis

Supplementary Table S2:

Table S3: Inflammatory markers detected in skin biopsies using the OLINK platform

	average						significance vs healthy	
	**healthy** n=13	**S.E.M.**	**AD** n=14	**S.E.M.**	**Pso** n=14	**S.E.M.**	**AD**	**Pso**
**IL-8**	4,87115	0,3411814	7,8491629	0,6214693	9,8821646	0,4795054	*	****
**VEGFA**	7,4924808	0,1314118	7,8791907	0,1473387	8,4142885	0,1565898		**
**CD8a**	4,3213	0,2052149	5,3767607	0,1730533	5,8116585	0,3742144	*	
**CCL7**	0,2	0	1,1083057	0,3231024	1,7868631	0,2938798		***
**GDNF**	0,6762015	0,2939994	1,5537171	0,3421446	0,9106731	0,3343769		
**CDCP1**	3,8168369	0,2732745	5,2258557	0,3570242	5,0280808	0,3050226		
**CD244**	2,4975638	0,2272778	3,1565393	0,105904	3,1023546	0,128266		
**OPG**	3,42887	0,0830172	4,8098071	0,1985826	4,0585862	0,1707877	****	
**LAP TGF-β1**	4,5240492	0,2310879	5,152935	0,1235138	4,93101	0,1240555		
**uPA**	6,73178	0,1197985	8,5316007	0,2192328	8,0182885	0,193338	****	***
**IL-6**	0,9536162	0,4138713	2,2253086	0,5816999	2,8539377	0,5285863		
**IL-17C**	0,6184331	0,2684672	2,5103821	0,2281336	4,4968731	0,3709684	***	****
**MCP-1**	8,3844115	0,1448951	9,9017521	0,1923907	9,4699508	0,2125932	****	*
**IL-17A**	1,1592854	0,4246022	1,9214957	0,5127678	4,4282392	0,5586363		**
**CXCL11**	2,9709523	0,1498499	3,565425	0,2254132	3,7894315	0,2994151		
**AXIN1**	4,03204	0,1698771	4,7557614	0,1754444	4,5599269	0,1715181		
**TRAIL**	3,4925808	0,1419211	4,3567421	0,1410367	4,28051	0,1497795	*	
**CXCL9**	4,0147523	0,2509781	5,0402971	0,2758142	6,4508015	0,4378604		**
**CST5**	0,9262238	0,1087625	0,9698793	0,1130671	1,0379162	0,1466543		
**IL-1α**	7,0631346	0,3448219	4,2599757	0,3306641	3,7016354	0,299551	*	****
**OSM**	1,8154362	0,3515998	3,7924557	0,4221498	3,9312715	0,2690013		**
**CXCL1**	5,6929177	0,1623545	7,6565314	0,3777267	10,24863	0,4897771	*	****
**CCL4**	2,1816115	0,4236418	3,501605	0,1642859	4,2992631	0,2230943		*
**CD6**	2,3194838	0,2900302	3,4406107	0,1645609	3,2054562	0,3255137		
**SCF**	3,3366977	0,075559	3,3734857	0,0563133	3,2281038	0,0450057		
**IL-18**	8,3317477	0,1899403	10,124829	0,2069151	10,109142	0,2470927	****	***
**TGF-α**	3,0531954	0,2236045	3,8780114	0,1710049	4,1612238	0,2450776		
**CCL13**	11,504585	0,1413005	13,593251	0,3321115	12,079608	0,3563326	***	
**CCL11**	2,6947038	0,4315224	2,63003	0,3978313	2,4394846	0,4710001		
**TNFSF14**	3,4328362	0,2922044	4,4962229	0,2119343	4,2921569	0,1487428		
**MMP-1**	7,3271915	0,3693112	10,367773	0,8070547	9,4121423	0,4418982		
**CCL19**	3,7255277	0,1801989	5,2388386	0,1975397	5,0604338	0,2504877	***	*
**IL-15RA**	1,4319569	0,2309661	2,2353629	0,0977878	2,00668	0,0510821		
**IL-10RB**	0,4431862	0,3006652	0,6266586	0,3467688	0,4417608	0,2997276		
**IL-18R1**	4,5696154	0,1460459	5,4794279	0,1327433	5,3259254	0,1763554	**	
**PD-L1**	1,4512546	0,3924693	3,45494	0,1065346	3,5921515	0,2185405	**	**
**CXCL5**	3,6688408	0,3568789	4,0024893	0,3291055	3,4796692	0,267538		
**RANKL**	1,2182446	0,2801675	2,7174364	0,2775991	2,0538623	0,245487	*	
**HGF**	8,4491085	0,1781602	8,5251171	0,1988418	8,5522608	0,1249108		
**IL-12B**	2,4722546	0,1154766	3,5190164	0,2699824	5,7179508	0,1801699		****
**IL-24**	1,0257223	0,377169	1,4989429	0,3750511	1,2240308	0,3848634		
**ARTN**	0,7703615	0,2834538	0,7155407	0,2784485	0,6316885	0,2744507		
**MMP-10**	3,9296177	0,2414588	5,5435721	0,4401425	3,9622762	0,3093402		
**TNF**	0,6677592	0,245382	1,6442514	0,2611889	2,6424846	0,272518		**
**CCL23**	4,8078215	0,14533	5,4117671	0,2542561	4,88182	0,2152548		
**CD5**	5,4503354	0,1313038	7,359835	0,2221299	7,1312485	0,3098307	****	**
**CCL3**	3,0317123	0,4039923	4,3731221	0,2085175	5,1097038	0,1479774		**
**Flt3L**	7,0817377	0,1647538	7,2900264	0,1991068	6,9359031	0,1541961		
**CXCL6**	3,7250023	0,1862251	4,4698636	0,2090742	5,07899	0,2509058		*
**CXCL10**	4,2646308	0,1972284	5,112865	0,2733655	6,4741608	0,4137262		**
**4E-BP1**	10,090469	0,1632298	10,59006	0,1264093	10,834172	0,0614818		*
**SIRT2**	3,9579646	0,5067966	5,0791979	0,4606963	4,8790808	0,1857896		
**CCL28**	2,7816308	0,0525492	2,7552543	0,0622024	2,7563931	0,0509712		
**DNER**	2,5004446	0,1259734	2,6495779	0,2109825	2,4454892	0,0621663		
**EN-RAGE**	1,9244492	0,3785893	3,7380314	0,5656862	4,2362623	0,5220453		

Continued next page

Supplementary Table S2:

Table S3: Inflammatory markers detected in skin biopsies using the OLINK platform (cont.)

	average						significance vs healthy	
	**healthy**<br>n=13	**S.E.M.**	**AD**<br>n=14	**S.E.M.**	**Pso**<br>n=14	**S.E.M.**	**AD**	**Pso**
**CD40**	10,159605	0,153704	11,428824	0,1490065	11,144778	0,1831521	***	*
**IL-33**	4,94531	0,1560157	5,319135	0,1133968	5,0154269	0,1092622		
**IFN-γ**	0,2	0	1,01642	0,5799593	5,1677185	0,5038504		****
**FGF-19**	1,4163069	0,2284458	1,4272421	0,2240136	1,7605346	0,0426088		
**LIF**	5,7074508	0,2023682	6,9466064	0,222835	5,7365731	0,1811798	*	
**MCP-2 CCL8**	4,97521	0,150175	5,9283129	0,2103297	6,0381577	0,2022495		*
**CASP-8**	3,8621331	0,0919633	4,65408	0,133365	4,5172746	0,0853155	**	**
**TNFRSF9**	1,8139562	0,414995	4,1795679	0,1842481	4,3459338	0,2071106	***	***
**TWEAK**	8,8847623	0,134235	8,1979321	0,2508856	8,21041	0,163202		
**CCL20**	1,9153715	0,3868829	4,1035943	0,3609067	6,1392738	0,4007403	*	****
**ST1A1**	4,6172446	0,1937477	4,9024286	0,1578613	4,5577769	0,138592		
**STAMBP**	6,8841254	0,1823071	7,5459193	0,1175322	7,1507508	0,1327303		
**ADA**	9,7523777	0,1586139	9,49763	0,1661539	9,8829315	0,147665		
**TNFB**	1,2420323	0,2867687	1,6819021	0,2601771	1,8457531	0,2377562		
**CSF-1**	6,1585869	0,1346276	6,5254293	0,1288751	6,3193162	0,0972728		

Supplementary Table S2:

Table S2: Antibodies used for MELC analysis

Target	Company	Clone number	Order number
CCR3-CD193	Biolegend	5E8	310706
**CCR4-CD194**	R&D System	205410	FAB1567P
CCR5-CD195	Biolegend	HEK/1/85a	313707
CCR6-CD196	Novus	MM0066-3L1	NBP2-12131PE
CCR7-CD187	R&D System	205410	FAB1567P
**CD1c**	Biolegend	L161	331505
CD3	Miltenyi Biotech	REA613	130-113-139
CD4	R&D System	A1161A1	357406
CD7	BD Pharmingen	M-T701	561604
CD8a	Biolegend	C8/144B	372904
**CD11b**	Beckman	Bear1	IM0530
**CD11c**	Miltenyi Biotech	REAL235	130-121-314
**CD14**	Biolegend	HCD14	325604
CD16	BD Pharmingen	3G8	561248
CD19	Biolegend	SJ25C1	363008
CD20	Bio-Rad	2H7	MCA1710A488T
CD27	Miltenyi Biotech	REA499	130-114-166
CD31	Bio-Rad	WM59	MCA1738F
CD45-RA	Miltenyi Biotech	REA562	130-113-365
CD45-RO	Bio-Rad	UCHL1	MCA461F
CD52	Bio-Rad	YTH34.5	MCA1642F
CD54	Invitrogen	HA58	12-0549-42
CD56	Biolegend	HCD56	318310
CD62L	Bio-Rad	FMC46	MCA1076F
CD62P	Biolegend	A4	304910
CD66b	Biolegend	G10F5	305104
CD94	Biolegend	REA787	305508
CD117	Miltenyi Biotech	REA787	130-111-593
**CD123**	Miltenyi Biotech	REA918	130-115-265
CD127	Biolegend	A019D5	351312
CD161	Miltenyi Biotech	REA631	130-113-595
**CD163**	BD Pharmingen	GHI/61	560933
CD183 (CXCR3)	Biolegend	G025H7	353708
CD184 (CXCR4)	Biolegend	12G5	306505
**CD206**	BD Biosciences	19.2	550889
Pan-cytokeratin	eBiosciences	AE1/AE3	53-9003-80
GRP15	Biolegend	Sa302A10	373003
**HLA-DR**	Miltenyi Biotech	AC122	130-113-398
Ki67	eBioscience	SolA15	12-5698-82
MPO	Abcam	EPR20257	ab225474
**Propidium Iodide**	Sigma-Aldrich		P4170

Markers depicted in bold were used for the principal component analysis of DC and macrophage subtypes

Supplementary Table S4:

Table S4: Markers used to identify T cell populations

T cell subtype	Marker				
CD8	$CD3^+$	$CD8^+$			
γδ	$CD3^+$	$CD4^-/CD8^-$	$CD161^-$		
Th22	$CD3^+$	$CD4^+$	$CCR4^+$		
Th17	$CD3^+$	$CD4^+$	$CCR4^+$	$CD161^+$	$CCR6^+$
Th1	$CD3^+$	$CD4^+$	$CCR5^+$		
Threg	$CD3^+$	$CD4^+$	$CCR6^-$		
Threg/DCs	$CD3^+$	$CD4^+$	$CD11c^+$		
Threg/Macophages	$CD3^+$	$CD4^+$	$CD14^+$		

Supplementary Table S5:

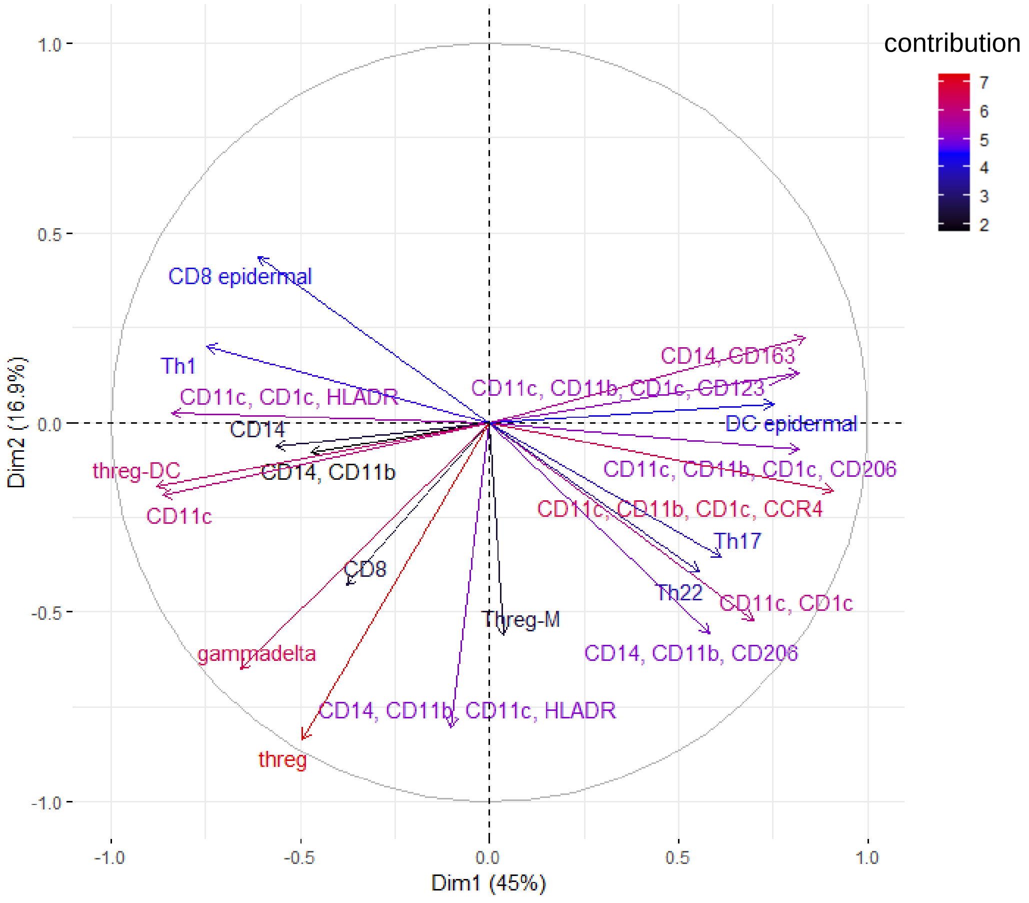

Figure S5: Principal component analysis based on the MELC analysis differentiates between Pso and AD patients. Contribution of each cluster to principal component analysis to the principal component analysis is shown for macrophage, DC and T cell populations as well as epidermal DC and CD8a^{+} T cell localization. Pso (n=5), AD (n=5).
